# Impact of RA treatment strategies on lipids and vascular inflammation in rheumatoid arthritis: a secondary analysis of the TARGET randomized active comparator trial

**DOI:** 10.1186/s13075-024-03352-3

**Published:** 2024-06-24

**Authors:** Katherine P. Liao, Pamela Rist, Jon Giles, Leah Santacroce, Margery A. Connelly, Robert J. Glynn, Paul Ridker, Ahmed Tawakol, Joan Bathon, Daniel H. Solomon

**Affiliations:** 1 Division of Rheumatology, Inflammation, and Immunity, Brigham and Women’s Hospital, 60 Fenwood Rd, Boston, MA 02115 USA; 2 Department of Medicine, Harvard Medical School, Boston, MA USA; 3 Division of Preventive Medicine, Brigham and Women’s Hospital, Boston, MA USA; 4 Division of Rheumatology, College of Physicians and Surgeons, Columbia University, New York, NY USA; 5 Labcorp, Morrisville, NC 27560 USA; 6 Cardiology Division, Massachusetts General Hospital, Boston, MA USA

**Keywords:** Rheumatoid arthritis, Lipids, Cardiovascular disease

## Abstract

**Background:**

Treatments for rheumatoid arthritis (RA) are associated with complex changes in lipids and lipoproteins that may impact cardiovascular (CV) risk. The objective of this study was to examine lipid and lipoprotein changes associated with two common RA treatment strategies, triple therapy or tumor necrosis factor inhibitor (TNFi), and association with CV risk.

**Methods:**

In this secondary data analysis of the TARGET trial, methotrexate (MTX) inadequate responders with RA were randomized to either add sulfasalazine and hydroxychloroquine (triple therapy), or TNFi for 24-weeks. The primary trial outcome was the change in arterial inflammation measured in the carotid arteries or aorta by FDG-PET/CT at baseline and 24-weeks; this change was described as the target-to-background ratio (TBR) in the most diseased segment (MDS). Routine lipids and advanced lipoproteins were measured at baseline and 24-weeks; subjects on statin therapy at baseline were excluded. Comparisons between baseline and follow-up lipid measurements were performed within and across treatment arms, as well as change in lipids and change in MDS-TBR.

**Results:**

We studied 122 participants, 61 in each treatment arm, with median age 57 years, 76% female, and 1.5 year median RA disease duration. When comparing treatment arms, triple therapy had on average a larger reduction in triglycerides (15.9 mg/dL, *p* = 0.01), total cholesterol to HDL-C ratio (0.29, p-value = 0.01), and LDL particle number (111.2, *p* = 0.02) compared to TNFi. TNFi had on average a larger increase in HDL particle number (1.6umol/L, *p* = 0.006). We observed no correlation between change in lipid measurements and change in MDS-TBR within and across treatment arms.

**Conclusions:**

Both treatment strategies were associated with improved lipid profiles via changes in different lipids and lipoproteins. These effects had no correlation with change in CV risk as measured by vascular inflammation by FDG-PET/CT.

**Trial registration:**

ClinicalTrials.gov ID NCT02374021.

**Supplementary Information:**

The online version contains supplementary material available at 10.1186/s13075-024-03352-3.

## Background

Lipids and lipoproteins are involved in the pathogenesis of cardiovascular disease (CVD), however in RA, the relationship between lipids and CV risk is more complex compared to the general population. Prior studies demonstrate that RA treatments modify lipids, in part by modifying disease activity and inflammation [1–4], which in turn modifies the relationship between lipid levels and CV risk. For example, in several studies, RA patients with the lowest low density lipoprotein cholesterol (LDL-C) levels appear to have the highest CV risk; it is possible that in patients with systemic inflammatory conditions relatively low LDL-C levels primarily reflect uncontrolled disease (e.g., RA) rather than CV risk as observed in the general population [5–8]. This complex relationship between CV risk and inflammation, RA treatments and lipids underscores the need for a more detailed understanding of how different RA disease modifying antirheumatic drugs (DMARDs) impact lipids and lipoprotein levels, and possibly CV risk.

Tumor necrosis factor inhibitors (TNFis) are the most prescribed biologic disease modifying anti-rheumatic drug (bDMARD) [9–11]. In observational studies, use of TNFi associates with reduced CV risk [12–14]. However, use of a TNFi also associates with increases in lipid parameters typically associated with increased CV risk. In a comparative effectiveness clinical trial of early RA patients, routinely checked lipids, such as total cholesterol (TC), LDL-C, and high-density lipoprotein cholesterol (HDL-C) were observed to increase across all treatment arms, including methotrexate (MTX) in combination with tumor necrosis factor inhibitor (TNFi), triple therapy (MTX, sulfasalazine (SSZ), and hydroxychloroquine (HCQ)), as well as MTX monotherapy [2]. In a follow-up analysis, no differences in lipid changes were observed across the 3 treatment arms, and pooling data from the 3 arms demonstrated an association between lower disease activity and increases in TC, LDL-C and HDL-C. These data suggest that control of disease activity is a major driver of these lipid changes and that the different treatments had a similar effect on lipids.

In addition to TC, LDL-C, and HDL-C, other lipid parameters may be important. In a retrospective cohort of patients with systemic inflammatory conditions (e.g., RA) who had triglycerides and TC measured before and after TNFi initiation, increases were observed in TG levels as well as TC [15]. Advanced lipoprotein measurements measured by nuclear magnetic resonance (NMR), have not been routinely pursued in RA cohorts, even though they can provide additional information on lipid atherogenicity. Apolipoprotein B (apoB), a more direct measurement of atherogenic lipid particles has the strongest association with CV outcomes compared to other lipid and lipoprotein measurements [16]. ApoA1 is the main protein component of HDL that promotes reverse cholesterol transport [17, 18]. Lipid subfractions such as LDL particle number (LDL-P) and size are sometimes considered in treatment decisions for some at-risk CVD patients in the general population, but these measurements have not been reported in a cohort with RA [18, 19]. Higher LDL-P, smaller LDL size, and a lower HDL particle number (HDL-P) are associated with higher CV risk [20, 21]. There are a paucity of studies employing NMR lipid measurements in RA with the majority comparing RA patients with controls, demonstrating a generally more atherogenic, e.g., higher levels of small LDL particles in RA from cross-sectional analyses [22, 23]. To our knowledge, no prior studies in RA have simultaneously measured NMR lipid measurements and CV risk.

We recently completed the TARGET trial which was a randomized active comparator trial among patients with RA who were MTX inadequate responders to either additionally receive a SSZ and HCQ, or TNFi [24, 25]. The objective of TARGET was to compare differences in arterial inflammation, a marker for CV risk, between the two treatment arms. We found that both treatments similarly reduced arterial inflammation assessed by FDG PET/CT^25^. Lipids and advanced lipoproteins parameters were measured prior to randomization and at study completion, after approximately 24 weeks of treatment. The objective of the present analysis was to determine whether lipid and lipoprotein changes differed between the two treatment strategies. Further, we determined differential changes in lipid and lipoprotein parameters were associated with different changes in CV risk as measured by arterial FDG PET/CT.

## Methods

### Study design and participants

The overall study design and the primary results of the TARGET study have been previously published [24, 25]. Briefly, the trial included 138 RA patients, men ≥45 years of age and women ≥50 years of age with a disease activity score 28 with CRP (DAS28-CRP) > 3.2 (moderate disease activity). All subjects must have been treated with MTX ≥15 mg once a week at a stable dose for ≥ 4 weeks prior to their screening visit (or ≥7.5 mg once a week for ≥8 weeks with documented intolerance). Use of low intensity statins, prednisone ≤10 mg once a day at a stable dose, and prior HCQ were allowed. Exclusion criteria included the use of a bDMARD or targeted synthetic DMARD (tsDMARD) in the 6 months prior to enrollment, or use of rituximab at any time prior to enrollment. Patients with prior CVD events were excluded. Specific to the current analyses, all patients on statins at their baseline visit were excluded. However, in contrast to the main trial, paired FDG PET/CT scans were not required for inclusion in this analysis; baseline and 24 week lipid and lipoprotein measurements were required.

### Procedures

Fasting blood samples were collected at the baseline visit prior to randomization to TNFi or triple therapy, and at their final visit at 24 weeks. Samples were measured for routine lipids, TC, LDL-C, HDL-C and TG at the clinical laboratory of Boston Children’s Hospital, Boston, MA. NMR measured lipoprotein parameters, specifically, LDL-P, LDL particle size, and HDL particles (HDL-P) were measured in EDTA plasma samples on Vantera® Clinical Analyzers (Labcorp, Morrisville, NC) using previously published methods [26]. Details of the lipoprotein classes, subclasses and sizes that were calculated using the latest LP4 deconvolution algorithm have been published previously [27]. These specific NMR measurements were selected as they are associated with increased risk independent of routine lipids, and are available in the clinic as an FDA approved test using NMR to assist with CV risk stratification [26]. NMR measurements were also obtained for apolipoprotein B (apoB), the main lipoprotein component of LDL-C, and considered a more accurate measurement of atherogenic lipids, and apoA1, the main lipoprotein component related to cholesterol efflux to HDL particles.

Participants underwent full-body [18]F-FDG-labelled PET/CT scanning at baseline and after 24 weeks. The primary outcome was the change in FDG uptake in either the aorta and carotid arteries between the two treatment groups at 24 weeks compared to baseline. This change was measured using the mean of the maximum target-to-background ratio in the most diseased segment (MDS TBR) of arterial [18]F-FDG uptake. Details on the imaging were provided in the main trial paper [24, 25].

### Clinical factors

At baseline, steroids and hydroxychloroquine (HCQ) use was assessed. A joint assessment was also performed to calculate the Disease Activity Score 28 (DAS28)-CRP at the baseline and 24-week visit. Blood samples were collected at those visits, concurrent with the FDG PET/CT.

### Statistical analysis

First, we compared baseline characteristics and CV risk factors between the two treatment arms. Next, we calculated the mean values and mean change over time in the routine lipids (TC, LDL-C, HDL-C, TG) and in advanced lipoprotein measurements (apolipoprotein (apo) B, LDL-P, LDL particle size, apoA1, HDL-P) separately for each treatment arm. Values greater than the mean plus 4 standard deviations or less than the mean minus 4 standard deviations for baseline, follow-up, or mean change were considered outliers and were set to missing. Additionally, quality control was performed and implausible values were set to missing. Using paired t-tests, we tested for significant changes in the routine lipids and advanced lipoprotein measurements between baseline and follow-up within the TNFi arm and triple therapy arm separately.

To determine whether changes in lipids differed by treatment arm, we constructed a linear regression model to test for association between treatment arm and change (baseline to 24 week) in lipid or lipoprotein parameter. The models were adjusted for age, sex, and the lipid or lipoprotein level at baseline, as well as stratification factors used in the main trial: steroid use at baseline and hydroxychloroquine use at baseline [25]. A second model included the aforementioned variables with the addition of change in DAS28-CRP to account for any changes in lipids due to RA disease activity and inflammation.

To test for correlation between lipid parameters and arterial inflammation as measured by MDS TBR, first we obtained Pearson correlations between baseline values of the routine lipid and lipoprotein values and baseline MDS TBR. Next, we tested for correlations between the change in lipids and lipoprotein values and change in MDS TBR. To explore whether treatment modified the association between routine lipids and advanced lipoproteins, we performed two sets of analyses. First, we stratified by treatment the correlations between the change in lipids and lipoprotein values with change in MDS TBR by treatment. Second, we included an interaction term between change in lipid or lipoprotein value and treatment assignment in separate linear regression models predicting change in MDS TBR. We also included terms for treatment assignment and change in lipid or lipoprotein value, age, sex, steroid use at baseline, and HCQ use at baseline.

### Role of the funding source

The funders of the study had no role in study design, data collection, data analysis, or data interpretation.

## Results

Among the 159 participants randomized into TARGET, first we excluded those with missing lipid measures at baseline or 24 weeks (*N* = 11). Next, we excluded the 26 individuals taking statins at baseline (*N* = 14 in the triple therapy and *N* = 12 in the TNFi arm). Therefore, the study population for these analyses is 122 individuals, with *n* = 61 in the MTX + TNFi and *n* = 61 in the triple therapy arm; median age was 57 years, 76% were female; and the median RA disease duration was 1.5 years (Table 1). The median weekly dose of MTX at baseline was 20 mg once a week. NSAID use at baseline was higher in the group randomized to TNFi compared to triple therapy. The mean baseline MDS TBR in this subgroup was 2.7 (SD 0.7).

**Table 1:** Characteristics of the TARGET population at baseline stratified by treatment arm

	All Patients (*n* = 122)	MTX + TNF inhibitor (*n* = 61)	Triple therapy (*n* = 61)	*P*-value
		*N* (%) or median (interquartile range)		
Age, years	57.0 (53.0, 63.0)	56.0 (53.0, 62.0)	58.0 (54.0, 63.0)	0.56
Sex, female	93 (76.2)	44 (80.3)	49 (80.3)	0.28
Race				
White	86 (74.8)	46 (80.7)	40 (69.0)	0.09
Black	18 (15.7)	9 (15.8)	9 (15.5)	
Other	11 (9.6)	2 (3.5)	9 (15.5)	
Ethnicity				
Hispanic	33 (27.0)	17 (27.9)	16 (26.2)	0.83
Non-Hispanic	89 (73.0)	44 (72.1)	45 (73.8)	
RA disease duration, years	1.5 (0.6, 5.9)	2.5 (0.6, 7.2)	1.4 (0.5, 5.3)	0.42
Serologic status, positive	78 (63.9)	39 (63.9)	39 (63.9)	1.00
DAS28-CRP	4.9 (4.0, 5.6)	5.0 (4.0, 5.5)	4.8 (4.0, 5.7)	0.86
hsCRP (mg/L)	4.9 (1.8, 11.3)	4.9 (1.6, 10.6)	5.1 (1.8, 11.3)	0.86
Glucocorticoid use	35 (28.7)	18 (29.5)	17 (27.9)	0.84
NSAID use	47 (38.5)	30 (49.2)	17 (27.9)	0.02
Aspirin use	24 (19.7)	16 (26.2)	8 (13.1)	0.19
Methotrexate weekly dose (mg)	20.0 (15.0, 25.0)	20.0 (15.0, 25.0)	20.0 (15.0, 25.0)	0.15
Heath assessment questionnaire	1.3 (0.6, 1.8)	1.3 (0.5, 1.9)	1.4 (0.8, 1.6)	0.86
Body mass index (kg/m2)	29.6 (26.5, 34.6)	29.7 (26.2, 34.5)	29.6 (26.5, 34.7)	0.99
High blood pressure	47 (38.5)	24 (39.3)	23 (37.7)	0.85
Hyperlipidemia	9 (7.4)	4 (6.6)	5 (8.2)	0.74
Diabetes mellitus	1 (0.8)	1 (1.6)	0	1.00
Tobacco use				
Current	14 (11.5)	5 (8.2)	9 (14.8)	0.48
Past	27 (22.1)	15 (24.6)	12 (19.7)	
Never	81 (66.4)	41 (67.2)	40 (65.6)	

IQR, inter-quartile range; RA, rheumatoid arthritis; IQR, interquartile range; hsCRP, high sensitivity C-reactive protein; NSAID, non-steroidal anti-inflammatory drugs. DAS28-CRP, disease activity score in 28 joints. *High intensity statin use was an exclusion. The inclusion of these two patients was considered a protocol violation and only determined after randomization and treatment initiation. **Cardiovascular risk factors include the presence of hypertension, diabetes, hyperlipidemia, or current smoking (all self-reported at baseline)

### Change in routine lipids within and across treatment arms at baseline and 24-week follow-up

No significant change in TC was observed in the triple therapy arm when comparing baseline and 24 weeks (Table 2A), while an 11.0 mg/dL increase on average (~ 5.4% change) (*p* = 0.002) was observed among those who initiated TNFi (Table 2B). When comparing across treatment arms, no significant difference in change was observed (Table 2C).

**Table 2:** Change in lipids at baseline before randomization and after 24 weeks^a^ within each treatment arm: (A) triple therapy, (B) TNFi; (C) comparison of changes in routine lipids between the two treatment arms

A. Triple therapy, *n*=61				
Lipids (mg/dL)	Baseline	24 weeks	Δ	*p*-value^b^
Total cholesterol	215.3 (38.8)	213.8 (38.2)	-1.5 (34.4)	0.73
LDL-C	112.4 (28.3)	112.3 (28.3)	-0.1 (25.0)	0.98
HDL-C	55.9 (18.2)	59.0 (17.8)	3.1 (10.7)	0.03
Triglycerides	119.1 (48.4)	108.5 (39.4)	-10.6 (40.9)	0.048
TC/HDL-C	3.9 (1.1)	3.6 (1.0)	-0.3 (0.7)	0.01

B. TNFi + MTX, *n*=61				
Lipids (mg/dL)	Baseline	24 weeks	Δ	*p*-value
Total cholesterol	204.6 (39.8)	214.7 (39.7)	11.0 (26.1)	0.002
LDL-C	108.0 (27.7)	113.8 (28.6)	5.8 (18.9)	0.02
HDL-C	55.0 (13.5)	57.9 (15.4)	2.9 (10.1)	0.03
Triglycerides	106.5 (44.4)	114.7 (49.9)	8.2 (33.8)	0.07
TC/HDL-C	3.9 (1.3)	3.9 (1.3)	0 (0.6)	0.86

C. Comparison of changes in lipids in between the treatment arms (TNFi vs. triple therapy)			
Lipids (mg/dL)	Beta^c^	95% CI	*p*-value
Total cholesterol	9.40	-0.91, 19.70	0.07
LDL-C	4.65	-2.92, 12.23	0.23
HDL-C	-0.10	-3.78, 3.57	0.96
Triglycerides	12.85	0.59, 25.12	0.04
TC/HDL-C	0.28	0.05, 0.50	0.02

^a^all lipids in mg/dL and reported as mean (SD); no subjects on statins

^b^all p-values from paired t-test

^c^Adjusted for age, sex, steroid use at baseline, HCQ use at baseline, and baseline lipid value

No significant change in LDL-C was observed in the triple therapy arm between baseline and 24 weeks (Table 2A); a 5.8 mg/dL increase on average (*p* = 0.02) (~ 5.4% change) was observed among those who initiated TNFi (Table 2B). The difference in change across the two treatment arms was not significant (Table 2C).

For HDL-C, subjects on triple therapy had a 3.1 mg/dL increase on average (*p* = 0.03) (Table 2A); a similar change was observed for subjects on TNFi (2.9 mg/dl increase on average; *p* = 0.03) (Table 2B). No significant difference in change was observed across treatment arms (Table 2C).

For TG, subjects on triple therapy had a 10.6 mg/dL reduction on average (*p* = 0.048), while no significant change was observed within the TNFi arm. In contrast to other routine lipids, subjects on triple therapy had a 12.9 mg/dL larger reduction in TG between baseline and 24 weeks compared to the TNFi arm, adjusting for age, sex, steroid use, HCQ use, and TG levels at baseline (*p* = 0.04); this association remained after adjusting for change in disease activity (*p* = 0.01) (Supplementary 1 A).

The TC/HDL-C ratio decreased in the triple therapy arm (*p* = 0.01) (Table 2A), while no change was observed in the TNFi arm (Table 2B) when comparing baseline with 24-week values. When comparing across treatment arms, those in the triple therapy arm had on average a 0.28 larger reduction in TC/HDL-C compared to TNFi and this association remained after additionally adjusting for change in disease activity (Table 2C).

### Change in advanced lipoprotein measures within treatments arms and across treatment arms at baseline and 24-weeks

Subjects initiating triple therapy had an average reduction of 5.4 mg/dL in apoB (*p* = 0.02) (Table 3A) while no change was observed among subjects initiating TNFi (Table 3A). No significant difference in change was observed across treatment arms (Table 3C).

**Table 3:** Advanced lipoprotein at baseline before randomization and after 24 weeks^a^ stratified by treatment arm: (A) triple therapy, (B) TNFi; (C) comparison of advanced lipoprotein measurements across treatment arms

A. Triple therapy, *n* = 61				
Lipids	Baseline	24 weeks	Δ	*p*-value^b^
ApoB, mg/dL	98.5 (16.4)	94.5 (18.3)	-5.4 (15.1)	0.02
LDL particle size, nm	21.1 (0.5)	21.2 (0.4)	0.1 (0.3)	0.004
LDL-P, nmol/L	1383.8 (317.7)	1308.5 (335.8)	-75.3 (270.8)	0.048
ApoA1, mg/dL	137.3 (25.9)	133.4 (24.4)	-3.9 (20.8)	0.19
HDL-P, umol/L	22.2 (3.9)	20.9 (4.0)	-1.2 (3.1)	0.01
apoB/apoA1	0.74 (0.21)	0.72 (0.19)	-0.02 (-0.06)	0.31

B. TNFi + MTX, *n* = 61				
Lipids	Baseline	24 weeks	Δ	*p*-value
ApoB, mg/dL	95.0 (15.3)	95.6 (16.7)	0.5 (12.1)	0.73
LDL particle size, nm	21.1 (0.4)	21.2 (0.4)	0 (0.3)	0.60
LDL-P, nmol/L	1296.1 (269.0)	1336.0 (344.7)	39.9 (197.6)	0.12
ApoA1, mg/dL	128.4 (21.4)	134.7 (22.1)	6.3 (19.0)	0.01
HDL-P, umol/L	20.1 (3.5)	21.3 (3.7)	1.2 (3.1)	0.004
apoB/ApoA1	0.75 (0.16)	0.72 (0.18)	-0.03 (0.06)	0.02

C. Comparison of changes in advanced lipoprotein measurements between treatment arms (TNFi vs. triple therapy).			
Lipids (mg/dL)	Beta	95% CI	*p*-value
ApoB, mg/dL	4.5	-0.3, 9.4	0.07
LDL particle size, nm	-0.08	-0.19, 004	0.20
LDL-P, nmol/L	102.6	14.2, 190.9	0.02
ApoA1, mg/dL	6.7	-0.5, 13.8	0.07
HDL-P, umol/L	1.7	0.6, 2.9	0.004
apoB/ApoA1	-0.01	-0.05, 0.04	0.76

^a^All lipids in mg/dL and reported as mean (SD); no subjects on statins

^b^all p-values from paired t-test

*Abbreviations* apoA1 = apolipoprotein A; apoB = apolipoprotein B; LDL-P = LDL particle number; HDL-P = HDL particle number; LDL = low density lipoprotein

A small (0.1 nm) increase in LDL particle size was observed in the triple therapy arm (*p* = 0.004) (Table 3A), while no change was observed in the TNFi arm (Table 3B). When comparing across treatment arms, no significant difference in change was observed (Table 3C).

An average reduction of 75.3 nmol/L in LDL-P was observed within the triple therapy arm (*p* = 0.048) (Table 3A) while no change was observed in the TNFi arm (Table 3B). When comparing across treatment arms, triple therapy on average had a small but significantly lower reduction in LDL-P (*p* = 0.02) (Table 3C), where lower LDL-P is generally associated with CV risk reduction.

No significant changes in apoA1 were observed within the triple therapy arm (Table 3A), however subjects initiating a TNFi had on average a 6.3 mg/dL increase (*p* = 0.01) (Table 3B). These differences within treatment arms did not translate to a significant difference in change of apoA1 across treatment arms (Table 3C).

The HDL-P concentration decreased on average by -0.02 umol/L in the triple therapy arm (*p* = 0.01) (Table 3A), while an increase of 1.2 umol/L on average was observed in the TNFi arm (*p* = 0.004) (Table 3B). When comparing across treatment arms, subjects on TNFi had on average small but larger increases in HDL-P compared to triple therapy (*p* = 0.004) (Table 3C). In summary, an increase in HDL-P generally associated with reduced CV risk were present in the TNFi but not triple therapy arm.

The apoB/apoA1 ratio did not change among subjects initiated on triple therapy (Table 3A) and decreased among those on TNFi (*p* = 0.02) (Table 3B). However, the change in ratio did not differ significantly across treatment arms (Table 3C).

### Correlations between change in routine lipids, advanced lipoproteins with MDS TBR

Of the 122 participants included in the main analyses, 90 had available information to study change in MDS TBR over time. In the main trial both treatment arms had significant reductions in the MDS TBR at baseline compared to follow-up with no differences between the two arms [25]. In the present study, we observed no significant correlations between the baseline measures of routine lipids and baseline MDS TBR, or between change in routine lipids and change in MDS TBR (Table 4A, 4B). Similarly, for advanced lipoproteins, no correlation was observed with baseline MDS TBR or between change in advanced lipoproteins and change in MDS TBR (Table 5A, 5B). When stratified by treatment, we observed no significant correlations between change in either routine lipids or advanced lipoproteins and change in MDS TBR (Supplemental Table 3). Additionally, our models found no significant interactions between change in routine lipids or advanced lipoprotein measurements and treatment arm (all p-values > 0.05; results not shown).

**Table 4:** (A) Baseline correlation between routine lipids (mg/dl) and baseline MDS TBR and (B) correlation between Δroutine lipids with ΔMDS TBR

A.	Correlation	*p*-value
TChol	0.02	0.83
LDL-C	0.09	0.42
HDL-C	-0.08	0.44
TG	-0.005	0.96
TC/HDL-C	0.10	0.36

B.	Correlation	*p*-value
ΔTChol	-0.07	0.49
ΔLDL-C	-0.13	0.23
ΔHDL-C	0.09	0.41
ΔTG	-0.06	0.58
ΔTC/HDL-C	-0.14	0.21

**Table 5:** (A) Baseline correlation between advanced lipids (mg/dl) and MDS TBR, and (B) correlation between Δroutine lipids with ΔMDS TBR

A.	Correlation	*p*-value
ApoB, mg/dL	0.13	0.22
LDL particle size, nm	0.02	0.89
LDL-P, nmol/L	0.13	0.24
ApoA1, mg/dL	-0.01	0.91
HDL-P, umol/L	0.03	0.75
apoB/ApoA1	0.09	0.38

B.	Correlation	*p*-value
**Δ**ApoB, mg/dL	-0.15	0.17
**Δ**LDL particle size, nm	0.09	0.42
**Δ**LDL-P, nmol/L	-0.14	0.20
**Δ**ApoA1, mg/dL	-0.04	0.74
**Δ**HDL-P, umol/L	-0.06	0.58
**Δ**apoB/ApoA1	-0.08	0.48

## Discussion

This study contributes several findings to our understanding of both routine lipid and advanced lipoprotein changes in two commonly used RA treatment strategies, triple therapy and methotrexate in combination with a TNFi. Prior studies have observed that RA disease activity and inflammation are likely an important driver of lipid changes [3, 28]. In this study, we demonstrate that the treatment strategies have modest but potentially differential effects on lipid changes independent of reducing inflammation after adjusting for disease activity. Specifically, MTX inadequate responders initiated on triple therapy had on average a larger reduction TC/HDL-C ratio, TG, and LDL-P compared to those initiated on TNFi; TNFi was associated with on average a modest but larger increase in HDL-P. Both treatments strategies were associated with modest changes towards lipid profiles associated with reduced CV risk, in different ways. Finally, we observed that the changes in lipids were not associated with differences in vascular inflammation as detected by FDG-PET/CT.

The secondary analyses on lipid data from TARGET have both similarities and differences from a previous post-hoc lipid analyses published from the TEAR study, an RA clinical trial with three arms: MTX monotherapy, triple therapy, and MTX + TNFi. The TEAR study observed an increase in TC, LDL-C and HDL-C at 24 weeks compared to baseline when comparing within each of the 3 treatment arms [2]; TG data were not available. In contrast, we observed an increase in HDL-C in the triple therapy arm and a significant increase in TC and LDL-C in the TNFi arm only. The magnitude of the changes in TARGET were smaller than those observed in TEAR. The difference in results may be in part explained by differences in the randomized populations. Since TARGET was designed with the primary purpose to study CV risk, all subjects were fasting at the time of the blood draw; for this lipid analysis, subjects on statin therapy were also excluded as statins are a potent LDL-C lowering agent. In contrast, TEAR, a comparative effectiveness RA treatment study, collected non-fasting samples with 10–15% of subjects on statin therapy. However, despite this difference, subjects in TARGET had a similar mean LDL-C level to subjects in TEAR. Additionally, a higher prevalence of diabetes was reported in TEAR, 79–87% compared to < 1% in TARGET; this low prevalence of diabetes in TARGET was a design choice since we excluded patients with prior CV events and those on high intensity statins. Thus, it is possible that both the difference in fasting status and the cardiometabolic profile of subjects may have resulted in different lipid changes associated with anti-inflammatory therapy. TARGET patients also had a shorter RA duration, mean 1.5 years compared to 3.5–5.1 years in TEAR; TEAR was published approximately 10 years ago, during a time period when early aggressive management for RA was being established [29]. These differences in RA duration and secular trends in management may explain the overall higher mean RA disease activity of DAS28-CRP 5.7 in TEAR compared to 4.9 in TARGET. Since the magnitude of change in inflammation is associated with changes in lipids, starting at a higher level of inflammation may explain the larger lipid changes observed in TEAR compared to TARGET.

All observed differences in the change in lipids and lipoproteins across treatments were modest. Subjects on triple therapy had on average a greater reduction in TC/HDL-C, TG, and LDL-P, all suggesting a less atherogenic profile. TNFi on average had a modest but larger increase in HDL-P compared to triple therapy, suggesting a greater effect of TNFi in increasing anti-atherogenic particles compared to triple therapy.

Clinical trials of therapies that raise HDL-C have not resulted in the large CV risk reduction that was anticipated based on data from large epidemiologic studies [30, 31]. Thus, interest has turned to alternative measurements that may better reflect the anti-atherogenic properties of HDL such HDL-P which is being considered as a marker for future therapies aimed at HDL [32]. HDL data were analyzed in TEAR; the post-hoc lipid analyses pooling data from all 3 treatment arms showed that the overall cohort had an improvement in HDL function, however the data could not be directly compared with TARGET since different lipid measurements were used, and differences across treatment arms was not tested [33].

The differences observed in lipids and lipoproteins between the two treatment groups in TARGET were not associated with differences in vascular inflammation. No correlation was observed between the change in lipids and lipoproteins with change in arterial inflammation. The few epidemiologic studies of changes in lipids versus change in arterial inflammation as measured by FDG PET CT in the general population support these findings. Lipid lowering interventions, including statins, LDL apheresis, and proprotein convertase subtilisin/kexin type 9 (PCSK9) antibody therapy, with their potent LDL-C lowering properties, are associated with a rapid dose dependent reduction in FDG uptake in arterial vessel walls [34, 35]. However, the reductions in LDL-C have not been shown to significantly associated with reduced arterial FDG update [36]. Thus, reductions in arterial inflammation achieved by lipid lowering interventions may be in part attributed to changes in the non-LDL fractions by inflammatory mediators that are also impacted by lipid lowering therapies [21, 37, 38].

We note limitations to this study. First, 33% of subjects at baseline were on low dose steroids, and steroid use can be associated with increases in TC and HDL-C [39, 40]. Patients on steroids could enter the study on no more than prednisone 10 mg daily or equivalent, and dose changes during the trial could not exceed prednisone 3 mg. Thus, changes in lipids related to steroid dosing changes were likely limited. Of note, the baseline prednisone usage was not statistically different in the two groups. To further address this concern, baseline steroid use was also included as a covariate in models comparing differences in lipid and lipoprotein changes across treatment groups. Vascular inflammation as measured by FDG PET CT may not be sensitive to CV risk changes associated with changes in lipids.

## Conclusions

Using data from one of the largest trials to date with detailed lipid and lipoprotein, treatment and CV imaging, we observed that two treatment strategies in RA, triple therapy and TNFi modify lipids and lipoproteins in different ways. Triple therapy had small but relatively larger effects for reducing the TC/HDL-C ratio, TG, LDL-P compared to TNFi. Meanwhile TNFi had modest but larger effects for increasing HDL particles compared to triple therapy. These differences exist after accounting for changes in RA disease activity, a known factor associated with changes in lipids. Finally, the differential effect of treatment strategies on lipids and lipoproteins did not translate to differences in vascular inflammation. Their effect is likely beneficial in reducing CV risk via different effects on lipid metabolism. With the multiple therapies available for the treatment of RA, these data can be taken into account when selecting a treatment strategy for RA patients with dyslipidemia. Alternatively, these data can shed light on the underlying lipid changes occurring among subjects already on an effective therapy for their RA to improve management of their lipid profiles and CV risk. In future studies, consideration can be given to performing similar lipid and lipoprotein analyses as a method to understand differences in CV safety signals arising from comparison trials of anti-inflammatory RA therapies.

### Electronic supplementary material

Below is the link to the electronic supplementary material.

Supplementary Material 1

## Data Availability

Data are in the process of uploaded to NIH dbGAP. In the interim, datasets used and/or analysed during the current study are available from the corresponding author on reasonable request and approval from the MGB IRB.

## Abbreviations

apoA1: apolipoprotein A1
apoB: apolipoprotein B
Bdmard: Biologic disease modifying anti-rheumatic drug
CRP: C-reactive Protein
CV: Cardiovascular
CVD: Cardiovascular disease
DAS28-CRP: Disease Activity Score 28 with C-reactive Protein
DMARDs: disease modifying antirheumatic drugs
EDTA: Ethylenediaminetetraacetic acid
FDA: Food and Drug Administration
FDG: [18 F]-fluorodeoxyglucose
FDG-PET/CT: [18 F]-fluorodeoxyglucose positron emission tomography/computed tomography
F-FDG: [18 F]-fluorodeoxyglucose
HCQ: Hydroxychloroquine
HDL: High-density lipoprotein
HDL-C: high-density lipoprotein cholesterol
HDL-P: HDL particle number
hsCRP: High-sensitivity C-reactive protein
LDL: Low-density lipoprotein
LDL-C: Low density lipoprotein cholesterol
LDL-P: LDL particle number
LP4: LipoProfile-4
MDS: Most diseased segment
MDS TBR: Target-to-background ratio in the most diseased segment
Mg: Milligrams
mg/dL: Milligrams per deciliter
MTX: Methotrexate
Nm: Nanometer
nmol/L: Nanomoles per liter
NMR: Nuclear magnetic resonance
NSAID: Non-steroidal anti-inflammatory drug
PCSK9: Proprotein convertase subtilisin/kexin type 9
RA: Rheumatoid arthritis
SD: Standard deviation
SSZ: Sulfasalazine
TARGET: Treatments Against RA and Effect on FDG-PET/CT
TBR: Target-to-background ratio
TC: Total cholesterol
TEAR: The Treatment of Early Aggressive Rheumatoid Arthritis Study
TG: Triglycerides
TNFi: tumor necrosis factor inhibitor
triple therapy: MTX, SSZ, and HCQ
tsDMARD: Targeted synthetic DMARD
umol/L: Micromole per litre
