# Supplementary material for: Impact of RA treatment strategies on lipids and vascular inflammation in rheumatoid arthritis: a secondary analysis of the TARGET randomized active comparator trial
Source: Arthritis Res Ther. 2024 Jun 24;26:123. doi: 10.1186/s13075-024-03352-3 (PMC11194931; doi:10.1186/s13075-024-03352-3)
Supplement: Supplementary file 1 — Supplementary Material 1 [file 13075_2024_3352_MOESM1_ESM.docx]

**Supplementary Table 1.** Association between randomized treatment group and a) Δtriglycerides and b) TC/HDL-C, adjusted by DAS28-CRP.

| **A.** | **Model 1** |  | **Model 2** |  |
| --- | --- | --- | --- | --- |
| **Variable** | **β (SE)** | **p-value** | **Beta (SE)** | **p-value** |
| Age | 0.5 (0.4) | 0.21 | 0.5 (0.4) | 0.23 |
| Female sex | -9.8 (7.3) | 0.18 | -8.5 (7.1) | 0.24 |
| ΔDAS28-CRP | - | - | 4.9 (2.2) | 0.03 |
| Steroid use at baseline | 11.2 (6.8) | 0.10 | 9.3 (6.7) | 0.17 |
| HCQ at baseline | 2.5 (14.1) | 0.86 | -1.2 (14.0) | 0.93 |
| TG at baseline | -0.4 (0.1) | <0.0001 | -0.4 (0.1) | <0.0001 |
| **TNFi (vs triple therapy)** | **12.9 (6.2)** | **0.04** | **15.9 (6.2)** | **0.01** |

| **B.** | **Model 1** |  | **Model 2** |  |
| --- | --- | --- | --- | --- |
| **Variable** | **β (SE)** | **p-value** | **Beta (SE)** | **p-value** |
| Age | 0.005 (0.007) | 0.50 | 0.005 (0.007) | 0.53 |
| Female sex | -0.08 (0.13) | 0.56 | -0.07 (0.13) | 0.59 |
| ΔDAS28-CRP |  |  | 0.04 (0.04) | 0.38 |
| Steroid use at baseline | 0.13 (0.13) | 0.31 | 0.12 (0.13) | 0.37 |
| HCQ at baseline | 0.31 (0.31) | 0.33 | 0.30 (0.31) | 0.34 |
| TC/HDL-C | -0.20 (0.05) | <0.001 | -0.20 (0.05) | <0.001 |
| **TNFi (vs triple therapy)** | **0.28 (0.11)** | **0.02** | **0.29 (0.12)** | **0.01** |

**Supplementary Table 2.** Association between randomized treatment group and differences in the change in lipoproteins, (A) LDL-P (C) HDL-P.

| **A.** | **Model 1** |  | **Model 2** |  |
| --- | --- | --- | --- | --- |
| **Variable** | **β (SE)** | **p-value** | **Beta (SE)** | **p-value** |
| Age | 1.3 (3.1) | 0.67 | 1.3 (3.1) | 0.69 |
| Female sex | -58.7 (50.7) | 0.25 | -55.3 (50.8) | 0.28 |
| ΔDAS28-CRP | - | - | 15.0 (15.6) | 0.34 |
| Steroid use at baseline | 43.2 (49.4) | 0.38 | 39.5 (49.6) | 0.43 |
| HCQ at baseline | -170.7 (107.7) | 0.12 | -185.9 (108.9) | 0.09 |
| LDL-P at baseline | -0.2 (0.1) | 0.03 | -0.2 (0.1) | 0.02 |
| **TNFi (vs triple therapy)** | **102.6 (44.6)** | **102.6 (44.60.02** | **111.2 (45.5)** | **0.02** |

| **B.** | **Model 1** |  | **Model 2** |  |
| --- | --- | --- | --- | --- |
| **Variable** | **β (SE)** | **p-value** | **Beta (SE)** | **p-value** |
| Age | -0.03 (0.04) | 0.004 | -0.03 (0.04) | 0.41 |
| Female sex | -0.2 (0.6) | 0.79 | -0.2 (0.6) | 0.74 |
| ΔDAS28-CRP | - | - | -0.2 (0.2) | 0.39 |
| Steroid use at baseline | 0.4 (0.6) | 0.47 | 0.5 (0.6) | 0.42 |
| HCQ at baseline | 0.9 (1.3) | 0.50 | 1.1 (1.4) | 0.44 |
| HDL-P at baseline | -0.3 (0.1) | 0.0002 | -0.3 (0.1) | 0.001 |
| **TNFi (vs triple therapy)** | **1.7 (0.6)** | **0.004** | **1.6 (0.6)** | **0.006** |

**Supplementary Table 3.** Correlation between change in advanced lipids with change in MDS TBR stratified by treatment.

|  | **Triple Therapy** | | **TNFi** | |
| --- | --- | --- | --- | --- |
|  | **Correlation** | **p-value** | **Correlation** | **p-value** |
| TC | -0.01 | 0.97 | -0.15 | 0.33 |
| LDL-C | -0.11 | 0.47 | -0.14 | 0.35 |
| HDL-C | 0.22 | 0.15 | -0.04 | 0.80 |
| TG | -0.06 | 0.69 | -0.03 | 0.83 |
| TC/HDL-C | -0.14 | 0.36 | -0.12 | 0.45 |
| ApoB mg/dL | -0.12 | 0.44 | -0.16 | 0.30 |
| LDL particle size | 0.12 | 0.43 | 0.02 | 0.88 |
| LDL-P (nmol/L) | -0.14 | 0.36 | -0.11 | 0.48 |
| ApoA1 mg/dL | -0.03 | 0.86 | -0.01 | 0.93 |
| HDL-P (umol/L) | -0.13 | 0.42 | 0.06 | 0.70 |
| apoB/ApoA1 | -0.03 | 0.84 | -0.16 | 0.29 |
